# Plantar fascia enthesopathy is highly prevalent in diabetic patients without peripheral neuropathy and correlates with retinopathy and impaired kidney function

**DOI:** 10.1371/journal.pone.0174529

**Published:** 2017-03-30

**Authors:** Francesco Ursini, Franco Arturi, Kassandra Nicolosi, Antonio Ammendolia, Salvatore D’Angelo, Emilio Russo, Saverio Naty, Caterina Bruno, Giovambattista De Sarro, Ignazio Olivieri, Rosa Daniela Grembiale

**Affiliations:** 1 Department of Health Sciences, University of Catanzaro “Magna Graecia”, Catanzaro, Italy; 2 Department of Medical and Surgical Sciences, University of Catanzaro “Magna Graecia”, Catanzaro, Italy; 3 Rheumatology Department of Lucania, San Carlo Hospital of Potenza and Madonna delle Grazie Hospital of Matera, Potenza, Italy; Weill Cornell Medical College in Qatar, QATAR

## Abstract

**Background:**

Aim of this study was to evaluate the prevalence of plantar fascia (PF) enthesopathy in Type 2 diabetes mellitus (T2DM) patients without distal peripheral neuropathy (DPN).

**Methods:**

We recruited 50 T2DM patients without DPN and 50 healthy controls. DPN was excluded using the Michigan Neuropathy Screening Instrument (MNSI). All patients underwent a bilateral sonographicevaluation of the enthesealportion of the PF.

**Results:**

PF thickness was significantly higher in T2DM patients (*p*<0.0001). T2DM patients presented a higher prevalence of entheseal thickening (*p* = 0.002), enthesophyte (*p* = 0.02) and cortical irregularity (*p* = 0.02). The overall sum of abnormalities was higher in T2DM patients (*p*<0.0001), as was the percentage of bilateral involvement (*p* = 0.005). In a logistic regression analysis, retinopathy predicted entheseal thickening (OR 3.5, *p* = 0.05) and enthesophytes (OR 5.13, *p* = 0.001); reduced eGFR predicted enthesophytes (OR 2.93, *p* = 0.04); body mass index (BMI) predicted cortical irregularity (OR 0.87, *p* = 0.05); mean glucose predicted enthesophyte (OR 1.01, *p* = 0.03); LDL cholesterol predicted cortical irregularity (OR 0.98, *p* = 0.02).

**Conclusions:**

Our data suggest that T2DM is associated with PF enthesopathyindependently of DPN.

## Introduction

Type 2 diabetes mellitus (T2DM) isan epidemic disease affecting about 9% of the adult population in Europe[1] and represents a major cause of disability,[2] mainly attributable to the development of microangiopathic and macroangiopathic vascular disease.[3] Beyond the classic complications including the diabetic foot, T2DM have been associated with an increased risk of several rheumatic conditions, i.e. Dupuytren's contracture, flexor tenosynovitis, adhesive capsulitis, diffuse idiopathic skeletal hyperostosis (DISH), crystal induced arthritis, rheumatoid arthritis, psoriatic arthritis and osteoarthritis;[4–6]for converse, other rheumatic diseases seem to be associated with a reduced risk of T2DM.[7] However, it is still a matter of debate if the susceptibility is attributable to the disease itself or if it is related to the most frequently associated risk factors (obesity, smoke or alcohol consumption).Metabolic disorders are known to alter the structure and the mechanical properties of tendons[8] and this phenomenon is of greater interest in T2DM, because of the role of tendons in diabetic foot biomechanics.[9]In the foot, Achilles tendon (AT), plantar fascia (PF) andmetatarso-phalangeal joints (MPJs) represent a complex biomechanical unit[10]and interact in the distribution of plantar pressure on the foot[11]by means of the Windlass mechanism, a key component of the normal gait.Both AT and PF abnormalities[12, 13]could contribute to the generation of augmented forefoot pressures observed in diabetic patients[14] thus increasing the risk of diabetic ulcerations.[15]For this reason, ATlengthening[16] and PF release[17]have been proposed for the management of recurrent diabetic ulcers.

In the last years, implementation of high-resolution ultrasonographic probes have greatly improved the ability to study superficial structures like tendons. Indeed, clinical studies suggest that ultrasonographic abnormalities are frequently detected in AT[18] and PF[19] from T2DM patients. Although less studied, the entheseal portion of the tendon, in particular, is a critical zone were the highest forces are applied, due to the sudden transition from soft tissue to bone.[20]Previous observations suggested that diabetic peripheral neuropathy (DPN) is the main contributor to the development of abnormal function[21]and structure[22]in PF from T2DM patients. However, other mechanisms could contribute to the development of such abnormalities. In a recent work[23], for example, our group demonstrated that AT enthesopathy is highly prevalent in T2DM patients in absence of DPN.

Therefore, aim of the present study was to evaluate the prevalence of asymptomatic sonographically-detected PF enthesopatic changes in T2DM patients without DPN and to evaluate the correlation of PF enthesopathy with disease features.

## Materials and methods

### Patients

All subjects included in the present study were recruited at the Diabetes Outpatient Clinic, University of Catanzaro “Magna Graecia”, Catanzaro, Italy.For inclusion in the present study, all consecutive patients with T2DM seenstarting from January 2016, in order to achieve a minimum number of 50 eligible patients. Out of 97 consecutive patients screened, 47 were excluded because refused to entry in the study or presented one or more exclusion criteria.

Exclusion criteria were predefined as follow: 1) diagnosis of Type 1 diabetes (T1DM);2) past diagnosis of DPN;3)past history of foot or ankle fracture or other injury; 4) past or current history of heel or other foot pain; 5) Michigan Neuropathy Screening Instrument (MNSI) score ≥ 7 in the questionnaire or ≥ 2.5 in the physical assessment.[24, 25] The MNSI is a widely used instrument for evaluating the risk of diabetic neuropathy with the highest sensitivity among other screening tests, about 75%[26].Written informed consent was obtained from all subjects involved in the present study. For comparison 50 age- and sex- matched healthy patients without diabetes were used.The study protocol was approved by the local Ethics Committee(Comitato Etico Azienda Ospedaliera Mater Domini, Catanzaro, Italy).

### Clinical assessment and anthropometric measurements

All patients underwent a careful medical history, including year of T2DM diagnosis, presence of comorbidities and current pharmacological treatments.

Height and weight were measured with patients wearing light clothing and no shoes, to the nearest 0.1 cm and 0.1 Kg respectively. Body Mass Index (BMI) was calculated with the standard formula: BMI = Weight (kg)/Height (m)^2^.

Systolic (sBP) and diastolic blood pressure (dBP) weremeasured on the left arm with the patient supine and after 5 minutes of rest.The presence of high blood pressure was recorded as a dichotomic variable if the patient: a) presented with a past diagnosis of high blood pressure; b) was taking antihypertensive medications at the time of observation; c) presented elevated blood pressure in at least two separate outpatient visits. The presence of macrovascular disease was recorded as a dichotomic variable if the patients had a past diagnosis of coronary artery disease (including myocardial infarction and angina), cerebrovascular disease (including stroke and transient ischemic attack) or peripheral arterial disease.

### Laboratory evaluation and evaluation of diabetic retinopathy

After overnight fasting, blood samples were obtained for laboratory evaluation. Plasma glucose, total cholesterol, high-density lipoprotein (HDL)-cholesterol, low-density lipoprotein (LDL)-cholesterol and creatininewere measured with automated chemistry analyzer (Cobas 6000/Cobas e411, Roche Diagnostics). Glycated hemoglobin (A1c) was measured by high-performance liquid chromatography (ADAMS A1c, HA-8180, Arkray). For analysis purpose, the last three values of fasting glucose and A1c obtained during the last year were used to calculate means.

Each patient was screened for the presence of diabetic retinopathy (DR) by an ophthalmologist with stereoscopic color fundusphotographsfollowing pupil dilationaccording to American Academy of Ophthalmology (AAO) recommendations.[27] The presence of DR was recorded as a dichotomic variable.

Kidney function was evaluated by estimated glomerular filtration rate (eGFR) using the CKD-EPI (ChronicKidneyDisease- Epidemiology Collaboration) equation[28]. Subjects with eGFR< 90 mL/minweredefinedashavingimpairedkidneyfunction.

### Ultrasonographic assessment

The ultrasonographic (US) assessment was performed at Rheumatology Research Unit, University of Catanzaro “Magna Graecia”, Catanzaro, Italy.

Before scanning, the patient was asked to lie down in prone position, leaving the foot hanging out the examination table.

Sonographic examination of the entheseal portion of PF was carried out by a physician experienced in musculoskeletal sonography (F.U.), blinded to the category of the patient, using a EsaoteMyLab 25 Gold machine equipped with a 6–18 MHz linear probe (Esaote LA435). Longitudinal andtransverse scans were performed, with the probe parallel and perpendicular, respectively, to the direction of PF fibers.

PF enthesisthickness was measured in longitudinal scans at the level where the PF fibers meet the medial calcaneal tuberosity.The prevalence of enthesopathy was defined as previously described,[29, 30] and elementary US findings (hypoechogenicity, entheseal thickening, calcification, enthesophytes, bony erosion, cortical irregularity, bursitis and tendon tear) were recorded as dichotomic variables.The presence of intra-tendinousor entheseal power-Doppler, a measure of inflammation[31], was also recorded.

### Statistical analysis

Data are expressed as mean ± standard deviation, median (25th–75th percentile), or number (percentage) as appropriate. Student’s t-test was used to compare means for continuous variables. The Fisher’s exact test was used to compare prevalences between dichotomous variables. The Pearson’s product-moment correlation coefficient and partial correlation analysis was used to evaluate correlation between continuous or categorical variables while logistic regression was used to calculate odds ratios (OR) for dichotomic dependent variables. Unless otherwise stated, data from both feet of all patients was used as a single independent observation. A *p*-value <0.05 was considered statistically significant. All tests were two-tailed. Interobserver agreement was estimated by using Cohen’s κ statistics. A κ value less than 0.20 was classified as poor agreement, between 0.21 and 0.40 fair agreement, between 0.41 and 0.60 moderate agreement, between 0.61 and 0.80 good agreement, and between 0.81 and 1 excellent agreement[32]. The Statistics Package for Social Sciences (SPSS for Windows, version 17.0, SPSS Inc., Chicago, IL, USA) was used for all analyses.

## Results

### Patients characteristics

Clinical characteristics of the study population are detailed in Table 1.

**Table 1:** General characteristics of the study population.

	T2DM (N = 50)	Controls (N = 50)	*P* value
**Males**, n (%)	27 (54)	25 (50)	.84
**Age**, years	59.1 ± 7.5	57.5 ± 8.7	.15
**T2DM duration**, years	9.9 ± 6.8	N.A.	N.A.
**BMI**, Kg/m^2^	28.9 ± 4.7	26.9 ± 3.7	.001
*BMI ≥ 30 Kg/m*^*2*^, *n (%)*	17 (34)	13 (26)	.51
**sBP**, mmHg	129.6 ± 15.4	130.5 ± 16.0	.68
**dBP**, mmHg	80.0 ± 10.8	82.0 ± 9.1	.15
*High blood pressure*, *n (%)*	29 (58)	19 (38)	.07
**Diabetic retinopathy**, n (%)	20 (40)	N.A.	N.A.
**Macrovascular disease**, n (%)	20 (40)	N.A.	N.A.
**Mean fasting glucose**, mg/dL	139.9 ± 38.7	N.A.	N.A.
**Mean A1c**, %	7.2 ± 1.3	N.A.	N.A.
**Total cholesterol,** mg/dL	173.4 ± 37.1	N.A.	N.A.
**HDL-cholesterol,** mg/dL	52.7 ± 13.6	N.A.	N.A.
**LDL-cholesterol,** mg/dL	101.5 ± 35.6	N.A.	N.A.
**Triglycerides,** mg/dL	116.0 ± 53.8	N.A.	N.A.
**Creatinine**, mg/dL	0.91 ± 0.21	N.A.	N.A.
**eGFR,** mL/min	80.9 ± 18.8	N.A.	N.A.
***eGFR ≤ 90*** *mL/min*,*n (%)*	31 (62)	N.A.	N.A.
**Uric acid**, mg/dL	5.2 ± 1.3	N.A.	N.A.
**Current treatment** • MNT, n (%) • OADs, n (%) • Insulin, n (%) • Statin, n (%)	5 (10) 42 (84) 3 (6) 36 (72)	N.A. N.A. N.A. N.A.	N.A. N.A. N.A. N.A.

**Legend:**T2DM, type 2 diabetes mellitus; BMI, body mass index; sBP, systolic blood pressure; dBP, diastolic blood pressure; A1c, glycated hemoglobin; HDL, high-density lipoprotein; LDL, low-density lipoprotein; eGFR, estimated glomerular filtration rate; MNT, medical nutrition therapy; OAD, oral anti-diabetic medications; N.A., not available. Data are presented as mean ± standard deviation, median (25th– 75th percentile), or number (percentage) as appropriate

As per intention, patients with T2DM and controls did not significantly differed in percentage of males (54% Vs 50%, *p* = 0.84) and age (59.1 ±7.5 years Vs 57.5 ± 8.7 years, *p* = 0.15).As expected, BMI was significant higher in T2DM patients although the prevalence of obese individuals (defined as having a BMI ≥ 30 Kg/m^2^) was not statistically different. Accordingly, all correlational analyses were conducted in separate models including BMI as a covariate.

Patients with T2DM had a meandisease duration of 9.9 ± 6.8 years, mean fasting glucose of 139.9 ± 38.7 mg/dL and mean A1c of 7.2 ± 1.3%. Out of 50 patients, 5 (10%) were treated with medical nutritional therapy (MNT) alone, 42 (84%) were treated with oral antidiabetic drugs (OADs) and 3 (6%) were treated with insulin. In addition, 36 (72%) patients were treated with statins.

### Interobserver agreement

Interobserver agreement was evaluated by Cohen’s κ statistics. Accordingly, a set of 40 randomly selected scans from study patients was evaluated by a second sonographer (C.B.) and κ values were calculated for each independent abnormality. Interobserver agreement was *good* for the detection of enthesophytes (κ = 0.67), *moderate* for the detection of hypoechogenicity (κ = 0.44) and entheseal thickening (κ = 0.54) and *fair* for the detection of cortical irregularities (κ = 0.36). Agreement for tears, erosions, calcifications, bursitis and power-Doppler signal was not calculated because no such abnormalities were detected in the evaluation series.

### Sonographic characteristics of PF enthesis in T2DM patients and controls

Comparative sonographic characteristics of PF enthesis are reported in Table 2.

**Table 2:** Prevalence of enthesopathic changes in plantar fascia from patients and controls.

	T2DM (N = 100)	Controls (N = 100)	*P* value
**PF enthesis thickness**, mm	3.7 ± 0.9	3.0 ± 0.6	< .0001
**Hypoechogenicity**, %	6	1	.12
**Thickening**, %	12	1	.002
**Calcification**, %	2	0	.50
**Enthesophyte**, %	28	14	.02
**Bony erosion**, %	0	0	1.00
**Bony irregularity**, %	19	7	.02
**Bursitis**, %	1	0	1.00
**Tear**, %	0	0	1.00
**Power-Doppler**, %	0	0	1.00
**Sum of abnormalities**, n	0 (0.5–0.9)	0 (0.1–0.3)	< .0001
**Bilateral involvement**, %*	16 (32)	4 (8)	.005

**Legend:** PF, plantar fascia; T2DM, type 2 diabetes mellitus. * Data are relative to the total patients number (not tendons).Data are presented as mean ± standard deviation, median (25th– 75th percentile), or number (percentage) as appropriate.

Thickness of PF enthesis was significantly higher in T2DM patients (3.7 ± 0.9 mm Vs 3.0 ± 0.6 mm, *p*<0.0001). T2DM patients presented a significantly higher prevalence of entheseal thickening (12% Vs 1%, *p* = 0.002), enthesophyte (28% vs14%, *p* = 0.02) and cortical irregularity (19% Vs 7%, *p* = 0.02). No statistically significant differences were found in the number of tendons presenting hypoechogenicity, calcification, bursitis and tears. Entheseal power-Doppler signal was not detected in any patient. The overall sum of abnormalities was significantly higher in T2DM patients [0 (0.5–0.9) Vs 0 (0.1–0.3), *p*<0.0001], as was the percentage of bilateral involvement (32% Vs 8%, *p* = 0.005).

### Correlation between T2DM-related variables and enthesopathy features

In univariate analysis (Table 3), PF enthesisthickness correlated significantly with BMI (*r* = 0.26, *p* = 0.009), mean A1c (*r* = 0.20, *p* = 0.05), total cholesterol (*r* = 0.24, *p* = 0.01) andLDL cholesterol (*r* = -0.30, *p* = 0.003). However, after correction for BMI, the correlation between PF enthesisthickness and mean A1c was lost (Table 3).

**Table 3:** Univariate and BMI-adjusted correlation analyses with plantar fascia enthesis thickness as dependent variable.

	Univariate		Corrected for BMI	
	R	*P* value	R	*P* value
**Age**	-0.06	.55	-0.05	.59
**T2DM duration**	0.05	.64	0.001	.99
**sBP**	0.09	.38	0.11	.28
**dBP**	0.06	.55	0.05	.59
**BMI**	0.26	.009	N.A.	N.A.
**Mean glucose**	0.09	.37	0.08	.45
**Mean A1C**	0.20	.05	0.14	.15
**Total cholesterol**	-0.24	.01	-0.27	.008
**HDL-cholesterol**	-0.03	.75	-0.02	.84
**LDL-cholesterol**	-0.30	.003	-0.32	.001
**Triglycerides**	0.04	.65	0.04	.66
**Creatinine**	-0.08	.43	-0.05	.63
**eGFR**	0.06	.55	0.03	.80
**Uric acid**	-0.08	.43	-0.09	.39

**Legend:**T2DM, type2diabetes mellitus; sBP, systolic blood pressure; dBP, diastolic blood pressure; BMI, body mass index; A1c, glycated hemoglobin;HDL, high-density lipoprotein; LDL, low-density lipoprotein; eGFR, estimated glomerular filtration rate.Data are presented as mean ± standard deviation, median (25th– 75th percentile), or number (percentage) as appropriate.

Logisticregressionanalysiswasconducted to predictenthesealPF abnormalitiesusingT2DM-related variablesaspredictors. To avoid model overfitting, onlythe abnormalities that were more prevalent in T2DM patients were used as dependent variables and single logistic regression models were built for each predictor (Table 4).

**Table 4:** Univariate logistic regression analysis with enthesis hypoechogenicity, thickening, enthesophyte, bony irregularity as dependent variables.

	Hypoechogenicity		Thickening		Enthesophyte		Bony irregularity	
		*P* value	O.R.(95% CI)	*P value*	O.R.(95% CI)	*P value*	O.R.(95% CI)	*P value*
	O.R.(95% CI)	*P* value	O.R.(95% CI)	*P* value	O.R.(95% CI)	*P* value	O.R.(95% CI)	*P* value
**Age**	1.05 (0.97–1.13)	.20	1.03 (0.96–1.10)	.41	1.01(0.96–1.06)	.77	0.97(0.91–1.02)	.26
**Sex**	1.56 (0.43–5.72)	.50	1.83(0.51–6.51)	.35	0.80(0.33–1.92)	.62	2.11(0.73–6.12)	.17
**T2DM duration**	1.06(0.97–1.16)	.21	1.07(0.98–1.17)	.13	1.02(0.96–1.09)	.47	0.94(0.87–1.02)	.15
**OADs treated**	2.03(0.24–17.04)	.51	1.08(0.95–1.24)	.21	0.59(0.19–1.82)	.36	1.78(0.37–8.57)	.47
**Insulin treated**	2.25(0.59–8.55)	.23	1.21(0.30–4.92)	.79	0.95(0.33–2.75)	.93	1.34(0.42–4.26)	.61
**Statin treated**	4.35(0.53–35.7)	.17	4.87(0.60–39.62)	.14	0.96(0.36–2.53)	.94	4.02(0.86–18.70)	.08
**Retinopathy**	1.94(0.55–6.85)	.30	3.5(0.98–12.5)	.05	5.13(2.0–13.15)	.001	0.64(0.22–1.85)	.41
**Macrovascular** **disease**	1.29(0.36–4.54)	.70	0.72(0.20–2.58)	.62	0.50(0.19–1.28)	.15	1.11(0.40–3.07)	.83
**Reduced eGFR**	0.47(0.13–1.65)	.24	0.84(0.25–2.86)	.78	2.93(1.06–8.10)	.04	0.62(0.23–1.70)	.62
**High blood pressure**	0.57(0.16–2.0)	.38	1.02(0.30–3.45)	.98	0.95(0.39–2.30)	.91	1.30(0.46–3.66)	.61
**BMI**	1.00(0.88–1.14)	.99	1.09(0.96–1.22)	.17	1.01(0.92–1.11)	.86	0.87(0.76–0.99)	.05
**Mean glucose**	1.00(0.99–1.02)	.58	0.99(0.97–1.01)	.39	1.01(1.00–1.03)	.03	1.00(0.99–1.02)	.54
**Mean A1c**	1.25(0.80–1.95)	.32	0.96(0.61–1.53)	.87	1.27(0.92–1.75)	.15	0.94(0.64–1.38)	.74
**Total cholesterol**	0.99(0.98–1.02)	.91	1.00(0.99–1.02)	.74	0.99(0.98–1.01)	.25	0.99(0.98–1.00)	.19
**LDL-cholesterol**	0.99(0.97–1.01)	.44	0.99(0.97–1.01)	.38	0.99(0.98–1.01)	.32	0.98(0.96–1.00)	.02

**Legend:** T2DM, type 2 diabetes mellitus; OADs, oral antidiabetic drugs; eGFR, estimated glomerular filtration rate; BMI, body mass index; A1c, glycated hemoglobin; LDL, low-density lipoprotein.

A second analysis, including BMI as covariate (Table 5), was repeated in order to ascertain the contribution of body weight mediating the observed correlations. Retinopathy predicted enthesealthickening (OR 3.5, *p* = 0.05) and enthesophytes (OR 5.13, *p* = 0.001) but, after correction for BMI, the relation with enthesealthickening was lost. Reduced eGFR predicted enthesophytes(OR 2.93, *p* = 0.04) also after correction for BMI. BMI predicted inversely cortical irregularity (OR 0.87,*p* = 0.05). Mean fasting glucose predicted slightly, although significantly, the presence of enthesophyte (OR 1.01,*p* = 0.03) also after correction for BMI. LDL cholesterol predicted inversely the presence of cortical irregularity (OR 0.98, *p* = 0.02) also after correction for BMI.

**Table 5:** Logistic regression analysis with enthesis hypoechogenicity, thickening, enthesophyte, cortical irregularity as dependent variables after correction for body mass index.

	Hypoechogenicity		Thickening		Enthesophyte		Bony irregularity	
	O.R.(95% CI)	*P* value	O.R.(95% CI)	*P* value	O.R.(95% CI)	*P* value	O.R.(95% CI)	*P* value
**Age**	1.05 (0.97–1.13)	.20	1.03 (0.96–1.11)	.35	1.01(0.96–1.06)	.77	0.97 (0.91–1.03)	.28
**Sex**	1.56 (0.43–5.72)	.50	1.81 (0.50–6.54)	.36	0.80 (0.33–1.91)	.61	2.32 (0.78–6.95)	.13
**T2DM duration**	1.06 (0.97–1.16)	.20	1.06 (0.97–1.16)	.18	1.02 (0.96–1.09)	.49	0.95 (0.88–1.04)	.28
**OADs treated**	2.07 (0.24–17.82)	.51	1.09 (0.95–1.22)	.24	0.57 (0.18–1.79)	.33	2.68 (0.51–14.06)	.24
**Insulin treated**	2.34 (0.60–9.22)	.22	0.96 (0.22–4.19)	.95	0.93 (0.31–2.76)	.90	1.79 (0.53–6.05)	.35
**Statin treated**	4.35 (0.53–35.73)	.17	4.86 (0.59–39.8)	.14	0.96 (0.36–2.53)	.93	4.21 (0.89–20.00)	.07
**Retinopathy**	1.98 (0.55–7.13)	.30	3.16 (0.87–11.55)	.08	5.35 (2.04–14.03)	.001	0.76 (0.25–2.27)	.62
**Macrovascular** **disease**	1.29 (0.36–4.59)	.69	0.79 (0.22–2.90)	.73	0.50 (0.19–1.29)	.15	1.01 (0.36–2.85)	.99
**Reduced eGFR**	0.45 (0.13–1.65)	.23	1.02 (0.28–3.67)	.98	3.15 (1.11–8.96)	.03	0.49 (0.17–1.42)	.19
**High blood pressure**	0.56 (0.16–1.99)	.37	0.93 (0.27–3.22)	.91	0.94 (0.38–2.29)	.89	1.89 (0.61–5.89)	.27
**Mean glucose**	1.01 (0.99–1.02)	.58	0.99 (0.97–1.01)	.32	1.01 (1.00–1.03)	.03	1.01	.45
**Mean A1c**	1.27 (0.81–1.98)	.30	0.86 (0.51–1.44)	.57	1.28 (0.92–1.78)	.15	1.01 (0.69–1.49)	.94
**Total cholesterol**	0.99 (0.98–1.02)	.91	1.01 (0.99–1.02)	.85	0.99 (0.98–1.01)	.25	0.99 (0.97–1.00)	.14
**LDL cholesterol**	0.99 (0.97–1.01)	.44	0.99 (0.97–1.01)	.32	0.99 (0.98–1.01)	.31	0.97 (0.95–0.99)	.008

**Legend:** T2DM, type 2 diabetes mellitus; OADs, oral antidiabetic drugs; eGFR, estimated glomerular filtration rate; A1c, glycated hemoglobin; LDL, low-density lipoprotein.

## Discussion

In the present study we demonstrated a significantly higher prevalence of sonographically-detected PF enthesopathic changesin asymptomatic T2DM patients in whose the presence of DPN was ruled out using the MNSI, a sensitive and validated screening instrument.[26]In details, T2DM patients presented a significantly higher prevalence of entheseal thickening,enthesophytes and cortical irregularity. In addition, the cumulative number of abnormalities and the percentage of bilateral enthesopathy were significantly higher in patients compared to controls, suggesting that a systemic mechanism, rather than a mechanical one,might underlie this association.

Radiographically-detected PF calcific enthesopathy(calcaneal spur)is a very common finding in adults, reported to be asymptomatically present in up to 60% of the general population aged 60–69 years, with higher figures in women than in men.[33]For converse, the prevalence of sonographically-detected PF enthesopathy in the general population has not been systematically investigated despite the wide diffusion of high-resolution ultrasound equipment have greatly improved the definition of peripheral tendon conditions. In T2DM patients, different studies demonstrated an elevated prevalence of AT tendinopathy[18, 19, 34] and enthesopathy[30], while less is known about PF pathology. Similarly to what we found in the present study, a significant increase in PF thickness have been previously reported in both T1DM[35]and T2DM[19, 36]; consistently with our results PF thickness was positively correlated with BMI suggesting that PF enlargement could represent a paraphysiological response to excessive mechanical load. For this reason, all analyses in our study have been corrected to account for BMI. Furthermore, in our study population, the most frequent sonographic abnormalitieswere enthesophytes and cortical irregularities. Both these abnormalities express the same phenomenon of calcification of insertional tendon fibers and remodeling of the cortical bone, that in bony irregularities don’t reach a sufficient grade of organization to depict a definite enthesophyte.[31]Enthesophyte formation is a complex process largely unknown, but physical factors such as longitudinal traction[37] and verticalcompression[38]havebeenhypothesized to play a pivotalrole. Calcaneal spurs, the radiographic reciprocal of enthesophytes, have been historically reported as highly prevalent in T2DM [39]andcorrelated to DPN [40] that exerts its unfavorable effect *via*a) the alteration of foot biomechanics and load distribution[22]and b) thinning of intrinsic foot muscles and plantar soft tissue.[41]Thus, our study have the major advantage of having excluded a priori patients with suspected DPN and opens new scenarios in the pathophysiology of T2DM-associated enthesopathy.Diverse other mechanisms can be hypothesized. In our study the prevalence of enthesophytes was significantly related to the presence of retinopathy and impaired kidney function, also after the influence of BMI was excluded. This findingseems to mirror recent data that demonstrated the ability of PF thickness measurements to predict the development of diabetic retinopathy and nephropathy[42, 43]suggesting that tendon evaluation could be an early marker of diabetic complications. In this case, the role of collagen glycation could be substantial. In rabbit, the glycation-induced collagen cross-linking is directly associated with an increased matrix stiffness and other mechanical attributes of the tendon.[44] In addition,high glucose concentration up-regulates the expression of metalloproteinasesMMP-9 and MMP-13 in tendon cells that exert a detrimental effect on tendon structure.[45]From the other side, hyperglycemia produces a reduction in proteoglycans levels related to decreased synthesis or sulfation of glycosaminoglycans, which may contribute to tendon pathology.[46]

Another possible mechanism of enthesopathy in T2DM could be related to microvasculardisease. All the enthesesare naturally poor vascularized structures[47] and consequently exceptionally susceptible to ischemia. Ischemia, moreover, has been evoked as a possible mechanism of calcific tendinopathy.[48]Abnormal microvascular function, as in the case of T2DM microangiopathy, could be the shared mechanism between retinopathy, impaired kidney function, and plantar fascia pathology.[47]

Surprisingly, we found a significant inverse correlation between PF thickness and total and LDL-cholesterol after adjusting for BMI. This finding, at first glance seems conflicting with literature evidence. Actually, lipid disorders and in particular familial hypercholesterolemia (FH) have been associated with AT pathology, namely tendinous xanthomatosis.[8]Moreover, in FH patients, AT thickness is increased[49]and correlates directly with LDL-cholesterol levels. This finding however has not been consistently confirmed in other hypercholesterolemic states[49], and xanthomas are only rarely encountered in tendons different from AT. Therefore, we feel that knowledge on lipid-associated AT disease could not be automatically transferred to PF and we speculate that other mechanisms could be evoked in this statistical correlation. The majority of patients in our study cohort were treated with statins that, according to recent evidence could affect tendon structure and function. In humans, statins treatment has been associated with a significant reduction in AT tendon thickness;[50] while in rats statins administration increases the risk of calcific tendinopathy.[51] Therefore, we theorize that statin treatment could mediate the inverse correlation between PF thickness and cholesterol levels in our study cohort.

In conclusion our data, although limited by the explorative nature and the relatively small number of individuals recruited, suggest that asymptomatic changes at the PF enthesisoccur frequently in patients with T2DM independently of the presence of moderate to severe DPN detected with the use of MNSI. A main limitation of this approach, however is that this instrument, although well-validated, is not sufficiently sensitive to detect mild or small fiber neuropathy that occurs early in the natural history of T2DM, even before the onset of retinopathy. [52] This characteristic could explain the discrepancy between the relatively high prevalence of retinopathy (40%) and the absence of neuropathy in our study population. Net of these limitations, our finding opens new scenarios in understanding the pathophysiology of T2DM-associated tendon and enthesealpathology and its potential consequences on foot biomechanics. However, further studies are needed to better ascertain the role of collagen glycation and/or microvascular disease in the development of such abnormalities.
